# Crystal structure of *cis*-bis­{4-phenyl-1-[(3*R*)-1,7,7-tri­methyl-2-oxobi­cyclo­[2.2.1]heptan-3-ylidene]thio­semicarbazidato-κ^3^ *O*,*N* ^1^,*S*}cadmium(II) with an unknown solvent mol­ecule

**DOI:** 10.1107/S2056989015021428

**Published:** 2015-11-21

**Authors:** Vanessa Senna Nogueira, Leandro Bresolin, Christian Näther, Inke Jess, Adriano Bof de Oliveira

**Affiliations:** a Escola de Química e Alimentos, Universidade Federal do Rio Grande, Av. Itália km 08, Campus Carreiros, 96203-900 Rio Grande–RS, Brazil; b Institut für Anorganische Chemie, Christian-Albrechts-Universität zu Kiel, Max-Eyth-Strasse 2, D-24118 Kiel, Germany; c Departamento de Química, Universidade Federal de Sergipe, Av. Marechal Rondon s/n, Campus, 49100-000 São Cristóvão–SE, Brazil

**Keywords:** crystal structure, *ONS*-thio­semicarbazone donor, camphor-thio­semicarbazone, cadmium-thio­semicarbazone complex

## Abstract

The reaction between the racemic mixture of the camphor-4-phenyl­thio­semicarbazone derivative and cadmium acetate dihydrate yielded the title compound, [Cd(C_17_H_20_N_3_OS)_2_]. The Cd^II^ ion is six-coordinated in a distorted octa­hedral environment by two deprotonated thio­semicarbazone ligands acting as an *O,N,S*-donor in a tridentate chelating mode, forming five-membered chelate rings. In the crystal, the mol­ecules are connected *via* pairs of N—H⋯S and C—H⋯S inter­actions, building centrosymmetric dimers. One of the ligands is disordered in the campher unit over two sets of sites with site-occupancy factors of 0.7 and 0.3. The structure contains additional solvent mol­ecules, which are disordered and for which no reasonable split model was found. Therefore, the data were corrected for disordered solvent using the SQUEEZE routine [Spek (2015 ▸). *Acta Cryst.* C**71**, 9–18] in *PLATON*. Since the disordered solvents were removed by data processing, and the number of solvent entities was a suggestion only, they were not considered in the chemical formula and subsequent chemical or crystal information.

## Related literature   

For one of the first reports of the synthesis of thio­semicarbazone derivatives, see: Freund & Schander (1902 ▸). For one example of camphor oxidation to 1,2-diketone, see: Młochowski & Wójtowicz-Młochowska (2015 ▸). For the synthesis and crystal structure of an octa­hedral Cd^II^ complex with a thio­semicarbazone derivative, see: Fonseca *et al.* (2012 ▸). For a review on the coordination chemistry of thio­semicarbazone derivatives, see: Lobana *et al.* (2009 ▸).
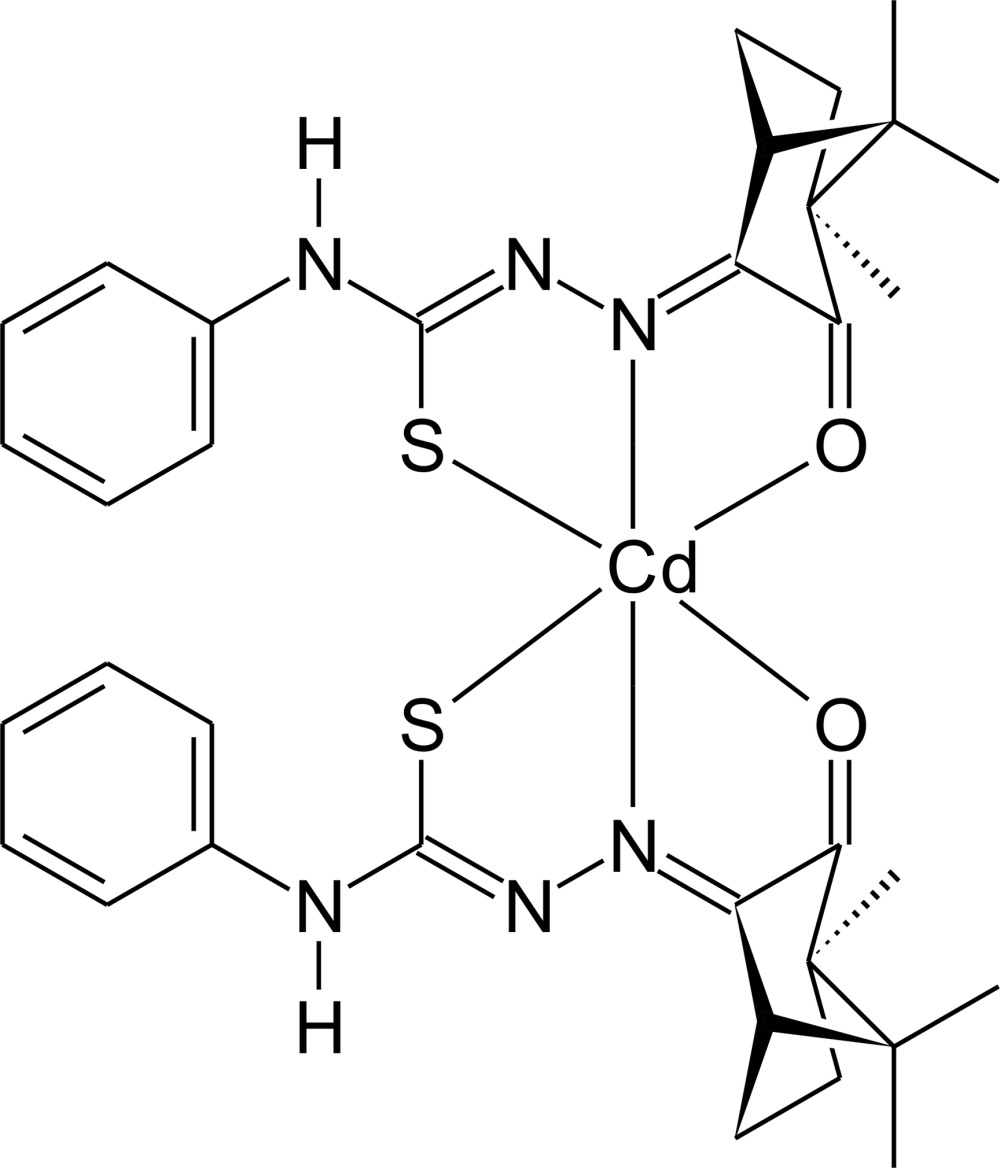


## Experimental   

### Crystal data  

[Cd(C_17_H_20_N_3_OS)_2_]
*M* *_r_* = 741.24
Triclinic, 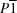

*a* = 10.3613 (3) Å
*b* = 12.3817 (4) Å
*c* = 16.5366 (6) Å
α = 68.727 (3)°
β = 72.094 (3)°
γ = 89.892 (3)°
*V* = 1866.74 (12) Å^3^
*Z* = 2
Mo *K*α radiation
μ = 0.73 mm^−1^
*T* = 170 K
0.18 × 0.14 × 0.08 mm

### Data collection  

Stoe IPDS-1 diffractometer
Absorption correction: numerical (*X-RED32* and *X-SHAPE*; Stoe & Cie, 2008 ▸) *T*_min_ = 0.831, *T*_max_ = 0.957
27175 measured reflections
8157 independent reflections
7089 reflections with *I* > 2σ(*I*)
*R*_int_ = 0.029

### Refinement  

*R*[*F* ^2^ > 2σ(*F* ^2^)] = 0.038
*wR*(*F* ^2^) = 0.103
*S* = 1.04
8157 reflections
439 parameters
20 restraints
H-atom parameters constrained
Δρ_max_ = 0.52 e Å^−3^
Δρ_min_ = −0.77 e Å^−3^

### 

Data collection: *X-AREA* (Stoe & Cie, 2008 ▸); cell refinement: *X-AREA*; data reduction: *X-AREA*; program(s) used to solve structure: *SHELXS97* (Sheldrick, 2008 ▸); program(s) used to refine structure: *SHELXL2014* (Sheldrick, 2015 ▸); molecular graphics: *DIAMOND* (Brandenburg, 2006 ▸); software used to prepare material for publication: *publCIF* (Westrip, 2010 ▸) and *enCIFer* (Allen *et al.*, 2004 ▸).

## Supplementary Material

Crystal structure: contains datablock(s) I, General. DOI: 10.1107/S2056989015021428/is5430sup1.cif

Structure factors: contains datablock(s) I. DOI: 10.1107/S2056989015021428/is5430Isup2.hkl

**Fig. 1.**
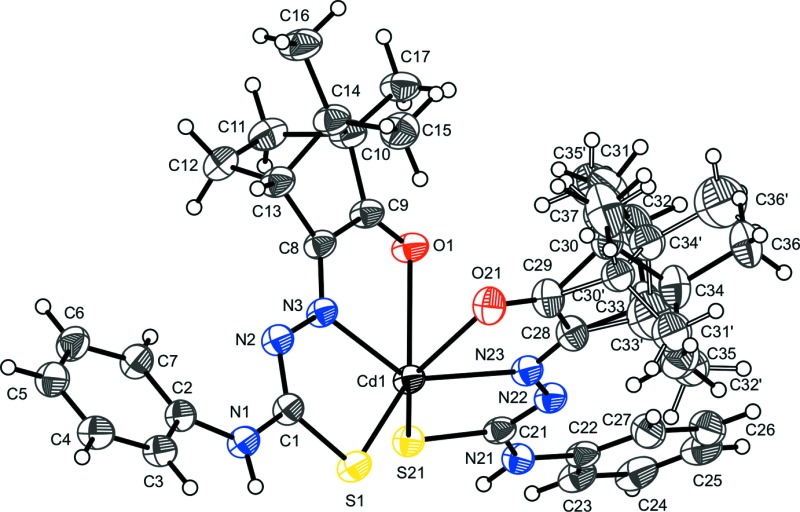
The molecular structure of the title compound with labeling and displacement ellipsoids drawn at the 30% probability level. Disorder is shown with full and open bonds.

**Fig. 2.**
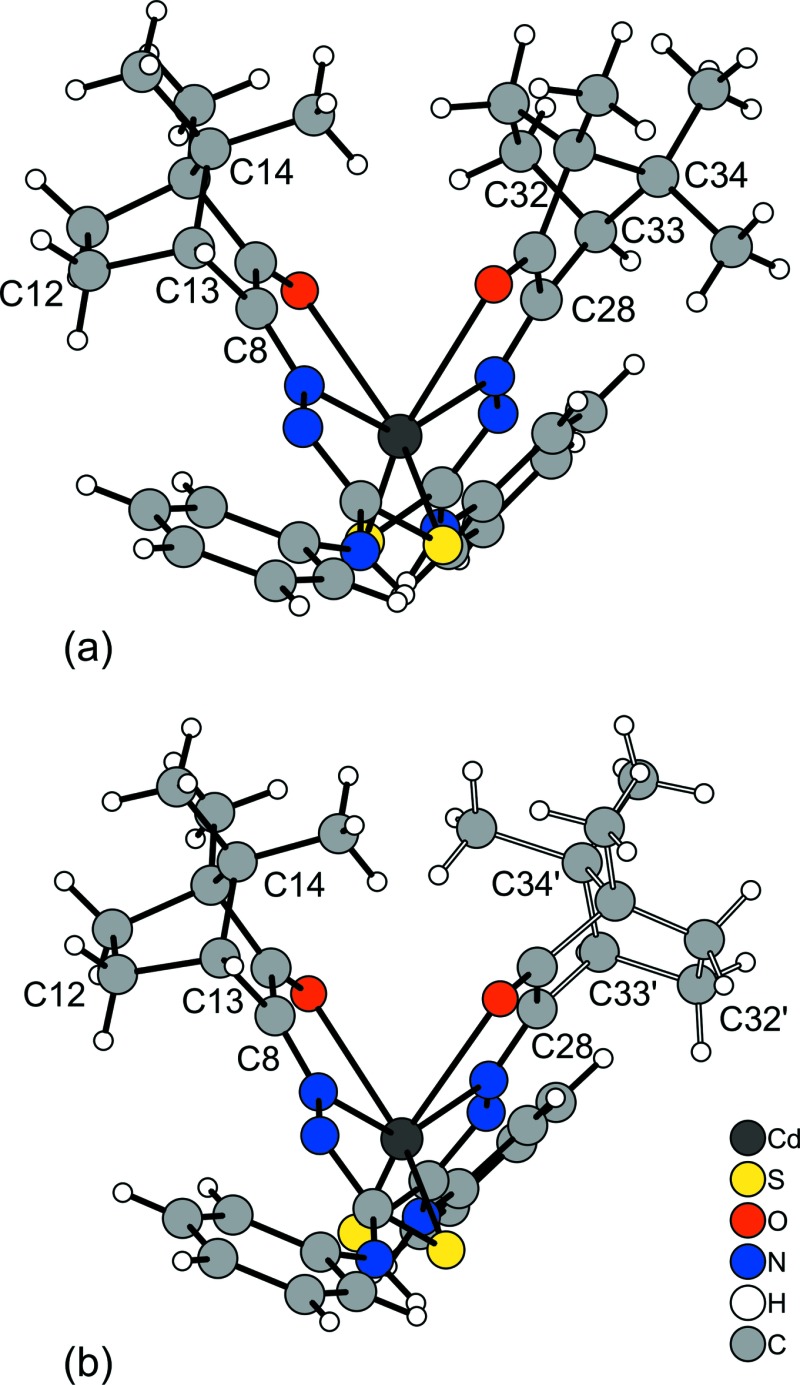
(a) Isotropic representation of the title compound with the disordered R-camphor entity. This ligand is labelled with C32, C33 and C34. (b) Isotropic representation of the title compound with the disordered S-camphor entity. This ligand is labelled with C32', C33' and C34'. The figure is valid for the asymmetric unit only and simplified for clarity.

**Fig. 3.**
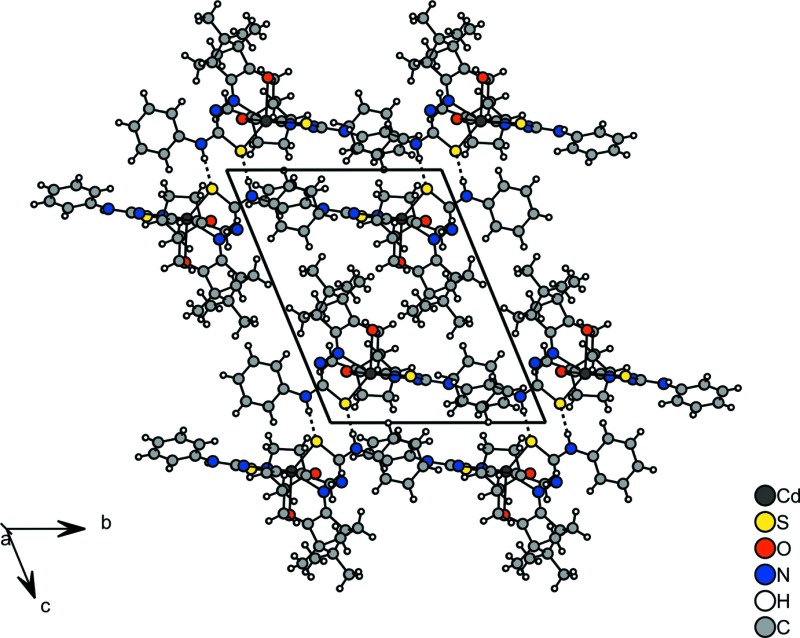
A packing diagram of the title compound viewed along the crystallographic a-axis, showing the N—H··· S hydrogen bonds (dashed lines). The C—H···S interactions are not shown for clarity. The disordered atoms are not shown. .

. DOI: 10.1107/S2056989015021428/is5430fig1.tif
The mol­ecular structure of the title compound with labeling and displacement ellipsoids drawn at the 30% probability level. Disorder is shown with full and open bonds.

a R b S . DOI: 10.1107/S2056989015021428/is5430fig2.tif
(*a*) Isotropic representation of the title compound with the disordered *R*-camphor entity. This ligand is labelled with C32, C33 and C34. (*b*) Isotropic representation of the title compound with the disordered *S*-camphor entity. This ligand is labelled with C32′, C33′ and C34′. The figure is valid for the asymmetric unit only and simplified for clarity.

a . DOI: 10.1107/S2056989015021428/is5430fig3.tif
A packing diagram of the title compound viewed along the crystallographic *a*-axis, showing the N—H⋯ S hydrogen bonds (dashed lines). The C—H⋯S inter­actions are not shown for clarity. The disordered atoms are not shown..

CCDC reference: 1436346

Additional supporting information:  crystallographic information; 3D view; checkCIF report

## Figures and Tables

**Table 1:** Hydrogen-bond geometry (Å, °)

*D*—H⋯*A*	*D*—H	H⋯*A*	*D*⋯*A*	*D*—H⋯*A*
N21—H21⋯S21^i^	0.88	2.58	3.363 (3)	148
C23—H23⋯S21^i^	0.95	2.97	3.629 (4)	128

Symmetry code: (i) 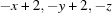.

## supplementary crystallographic information

### S1. Structural commentary

Our ongoing research deals with the synthesis and crystal structure analysis of thio­semicarbazone derivatives from natural products with an supra­molecular approach. Herein we report the synthesis and the crystal structure of a new Cd^II^ complex with the *R,S*-camphor-4-phenyl­thio­semicarbazone, a derivative from a racemic mixture of camphor. In the title compound the molecular structure matches the asymmetric unit and the metal ion is six-coordinated in a distorted o­cta­hedral environment by two thio­semicarbazonate ligands (Fig. 1). The ligands are *ONS*-donors and build a chelate coordination mode, where each ligand forms two five-membered rings. The maximum deviation from the mean plane of the Cd1/S1/C1/N2/N3/C8/C9/O1 chelating group amounts to 0.0811 (11) Å for S1 and for the Cd1/S21/C21/N22/N23/C28/C29/O21 chelating group amounts to 0.0801 (26) Å for C29, with the dihedral angle between the two chelate entities being measured as 73.16 (5)°. The two ligands are deprotonated and the negative charge is delocalized over the C—N—N—C—S fragment as suggested by their inter­mediate bond distances. The imine and thio­amide C—N distances indicate considerable double bond character, while the C—S distance is consistent with increased single bond character. This change on the bond character is a key feature to distinguish neutral/free or deprotonated/coordinated thio­semicarbazones. For the title compound, these distances are C8—N3 = 1.280 (3) Å, N2—N3 = 1.362 (3) Å, N2—C1 = 1.319 (3) Å and C1—S1 = 1.734 (3) Å for one ligand and C28—N23 = 1.278 (4) Å, N22—N23 = 1.367 (3) Å, N22—C21 = 1.313 (4) Å and C21—S21 = 1.743 (3) Å for the another one. The bond distances and the meridional coordination geometry agree with a similar Cd^II^ thio­semicarbazonate o­cta­hedral complex (Fonseca *et al.*, 2012) and are supported by literature data (Lobana *et al.*, 2009). The camphor molecule has two chiral carbon atoms and a racemic mixture was used in the synthesis.

From the two crystallographically independent ligands in the asymmetric unit, one is disordered in the campher unit with S. O. F. = 0.7:0.3 (Fig. 2). The complex molecules are connected into centrosymmetric dimers *via* pairs of N—H···S and C—H···S inter­molecurar inter­actions. The dimers are stacked along the crystallographic *a*-direction (Fig. 3 and Table 1).

### S2. Synthesis and crystallization

Starting materials were commercially available and were used without further purification. An *R,S*-camphor racemic mixture was oxidized with SeO_2_ to the respective 1,2-diketone (Młochowski & Wójtowicz-Młochowska, 2015). The synthesis of the *R,S*-camphor-4-phenyl­thio­semicarbazone derivative was adapted from a procedure reported previously (Freund & Schander, 1902). The ligand (2 mmol) was dissolved in ethanol (20 mL) and deprotonated with 1 mL of a 1 M KOH aqueous solution. Stirring was maintained for 40 min, while the reaction mixture turns yellow. A solution of cadmium acetate dihydrate (1 mmol) also in ethanol (20 mL) was added under continuous stirring and under slight warming to 333 K. After 3 h a yellow solid was formed. This solid was filtered-off, washed with small portions of cool ethanol and dried at room conditions. A bulk, rough material was observed and it was impossible to isolate enough qu­anti­ties of the title compound for complementar analysis or for yield calculation. Colourless crystals of the complex, suitable for X-ray analysis, were obtained by recrystallization from an ethanol solution.

### S3. Refinement

All non-hydrogen atoms except the disordered C atoms of lower occupancy were refined anisotropic. The C—H and N—H H atoms were positioned with idealized geometry and were refined isotropic with *U*_iso_(H) = 1.2 *U*_eq_(C,N) (1.5 for methyl H atoms) using a riding model.

The campher unit in one of the two independent ligands is disordered. This part was refined using a split model with S. O. F. = 0.7:0.3 and with similarity restraints (*SAME*). The site occupation factors were selected in order that the disordered atoms exhibits similar isotropic displacement parameters based on the isotropic refinement. If the isotropic displacement parameters are fixed and the S. O. F. is refined, similar values are obtained. Finally, the disordered atoms of higher occupancy were refined anisotropic.

The refined structure contained additional disordered solvate molecules. Because no reasonable split model was found, the data were corrected for disordered solvent using the *SQUEEZE* option in *PLATON* (Spek, 2015). The void volume and void count electrons amount to 234 Å^3^ and 55 e^-^·Å^-3^. The void electrons count of 55 can be assigned to two solvent ethanol molecules (52 electrons in total). Ethanol was the synthesis solvent. Since the disordered solvents were removed by data processing, and the estimated number of two ethanol molecules was a suggestion only, they were not considered in the chemical formula and subsequent chemical or crystal informations.

### Crystal data

**Table:**

[Cd(C_17_H_20_N_3_OS)_2_]	*V* = 1866.74 (12) Å^3^
*M**_r_* = 741.24	*Z* = 2
Triclinic, *P*1	*F*(000) = 764
*a* = 10.3613 (3) Å	*D*_x_ = 1.319 Mg m^−^^3^
*b* = 12.3817 (4) Å	Mo *K*α radiation, λ = 0.71073 Å
*c* = 16.5366 (6) Å	µ = 0.73 mm^−^^1^
α = 68.727 (3)°	*T* = 170 K
β = 72.094 (3)°	Block, colourless
γ = 89.892 (3)°	0.18 × 0.14 × 0.08 mm

### Data collection

**Table:**

Stoe IPDS-1 diffractometer	7089 reflections with *I* > 2σ(*I*)
Radiation source: fine-focus sealed X-ray tube, Stoe IPDS-1	*R*_int_ = 0.029
φ scans	θ_max_ = 27.0°, θ_min_ = 1.4°
Absorption correction: numerical (*X-RED32* and *X-SHAPE*; Stoe & Cie, 2008)	*h* = −13→13
*T*_min_ = 0.831, *T*_max_ = 0.957	*k* = −15→15
27175 measured reflections	*l* = −21→21
8157 independent reflections	

### Refinement

**Table:**

Refinement on *F*^2^	Secondary atom site location: difference Fourier map
Least-squares matrix: full	Hydrogen site location: inferred from neighbouring sites
*R*[*F*^2^ > 2σ(*F*^2^)] = 0.038	H-atom parameters constrained
*wR*(*F*^2^) = 0.103	*w* = 1/[σ^2^(*F*_o_^2^) + (0.0619*P*)^2^ + 0.5654*P*] where *P* = (*F*_o_^2^ + 2*F*_c_^2^)/3
*S* = 1.04	(Δ/σ)_max_ = 0.018
8157 reflections	Δρ_max_ = 0.52 e Å^−^^3^
439 parameters	Δρ_min_ = −0.77 e Å^−^^3^
20 restraints	Extinction correction: *SHELXL2014* (Sheldrick, 2015), Fc^*^=kFc[1+0.001xFc^2^λ^3^/sin(2θ)]^-1/4^
Primary atom site location: structure-invariant direct methods	Extinction coefficient: 0.0021 (6)

### Special details

**Table:**

Geometry. All e.s.d.'s (except the e.s.d. in the dihedral angle between two l.s. planes) are estimated using the full covariance matrix. The cell e.s.d.'s are taken into account individually in the estimation of e.s.d.'s in distances, angles and torsion angles; correlations between e.s.d.'s in cell parameters are only used when they are defined by crystal symmetry. An approximate (isotropic) treatment of cell e.s.d.'s is used for estimating e.s.d.'s involving l.s. planes.

### Fractional atomic coordinates and isotropic or equivalent isotropic displacement parameters (Å^2^)

**Table:**

	*x*	*y*	*z*	*U*_iso_*/*U*_eq_	Occ. (<1)
Cd1	0.63629 (2)	0.72196 (2)	0.19359 (2)	0.05151 (8)	
S1	0.73767 (8)	0.53984 (6)	0.18346 (6)	0.06243 (19)	
O1	0.42054 (19)	0.82822 (16)	0.20261 (16)	0.0612 (5)	
N1	0.6277 (3)	0.37580 (19)	0.15356 (18)	0.0580 (6)	
H1	0.7055	0.3510	0.1589	0.070*	
N2	0.4807 (2)	0.51031 (18)	0.17160 (17)	0.0540 (5)	
N3	0.4666 (2)	0.61471 (18)	0.18144 (16)	0.0497 (5)	
C1	0.6024 (3)	0.4765 (2)	0.16824 (19)	0.0523 (6)	
C2	0.5488 (3)	0.3049 (2)	0.1312 (2)	0.0537 (6)	
C3	0.5770 (3)	0.1895 (2)	0.1500 (2)	0.0576 (6)	
H3	0.6405	0.1606	0.1813	0.069*	
C4	0.5128 (3)	0.1175 (3)	0.1231 (3)	0.0676 (8)	
H4	0.5329	0.0393	0.1357	0.081*	
C5	0.4201 (3)	0.1579 (3)	0.0783 (3)	0.0709 (8)	
H5	0.3767	0.1082	0.0596	0.085*	
C6	0.3904 (4)	0.2709 (3)	0.0608 (3)	0.0706 (8)	
H6	0.3253	0.2984	0.0307	0.085*	
C7	0.4542 (3)	0.3452 (3)	0.0866 (2)	0.0648 (7)	
H7	0.4334	0.4232	0.0737	0.078*	
C8	0.3484 (3)	0.6498 (2)	0.1934 (2)	0.0527 (6)	
C9	0.3312 (3)	0.7644 (2)	0.2025 (2)	0.0552 (6)	
C10	0.1844 (3)	0.7820 (3)	0.2097 (2)	0.0646 (7)	
C11	0.1820 (4)	0.8005 (3)	0.1098 (3)	0.0748 (9)	
H11A	0.0954	0.8285	0.1018	0.090*	
H11B	0.2593	0.8582	0.0620	0.090*	
C12	0.1945 (4)	0.6810 (4)	0.1038 (3)	0.0788 (9)	
H12A	0.2754	0.6837	0.0517	0.095*	
H12B	0.1116	0.6514	0.0967	0.095*	
C13	0.2109 (3)	0.6035 (3)	0.1985 (2)	0.0639 (7)	
H13	0.1940	0.5171	0.2169	0.077*	
C14	0.1155 (3)	0.6547 (3)	0.2631 (3)	0.0679 (8)	
C15	0.1274 (4)	0.6047 (4)	0.3600 (2)	0.0842 (10)	
H15A	0.2236	0.6137	0.3555	0.126*	
H15B	0.0750	0.6469	0.3966	0.126*	
H15C	0.0912	0.5217	0.3896	0.126*	
C16	−0.0346 (3)	0.6409 (4)	0.2700 (3)	0.0880 (11)	
H16A	−0.0889	0.6755	0.3122	0.132*	
H16B	−0.0432	0.6806	0.2091	0.132*	
H16C	−0.0680	0.5577	0.2931	0.132*	
C17	0.1301 (3)	0.8764 (3)	0.2432 (3)	0.0763 (9)	
H17A	0.0347	0.8809	0.2455	0.114*	
H17B	0.1354	0.8583	0.3048	0.114*	
H17C	0.1849	0.9515	0.2012	0.114*	
S21	0.80796 (7)	0.89648 (6)	0.07495 (5)	0.05587 (16)	
O21	0.4762 (2)	0.62965 (17)	0.36571 (15)	0.0656 (5)	
N21	0.9069 (2)	1.0676 (2)	0.10330 (18)	0.0576 (5)	
H21	0.9552	1.0802	0.0460	0.069*	
N22	0.7536 (2)	0.9355 (2)	0.23571 (18)	0.0559 (5)	
N23	0.6675 (2)	0.83345 (19)	0.27396 (17)	0.0541 (5)	
C21	0.8192 (3)	0.9663 (2)	0.1475 (2)	0.0533 (6)	
C22	0.9341 (3)	1.1559 (3)	0.1333 (2)	0.0608 (7)	
C23	1.0073 (3)	1.2596 (3)	0.0638 (3)	0.0693 (8)	
H23	1.0365	1.2659	0.0018	0.083*	
C24	1.0382 (4)	1.3540 (3)	0.0841 (4)	0.0852 (12)	
H24	1.0886	1.4242	0.0362	0.102*	
C25	0.9959 (4)	1.3452 (4)	0.1731 (4)	0.0950 (14)	
H25	1.0156	1.4097	0.1874	0.114*	
C26	0.9245 (4)	1.2426 (4)	0.2422 (4)	0.1024 (16)	
H26	0.8959	1.2371	0.3041	0.123*	
C27	0.8932 (3)	1.1461 (4)	0.2232 (3)	0.0841 (11)	
H27	0.8447	1.0755	0.2714	0.101*	
C28	0.5920 (3)	0.8037 (2)	0.3577 (2)	0.0616 (7)	
C29	0.4897 (4)	0.6983 (3)	0.4008 (2)	0.0657 (7)	
C30	0.3914 (5)	0.7073 (4)	0.4896 (3)	0.0636 (10)	0.7
C31	0.3272 (7)	0.8220 (6)	0.4572 (4)	0.091 (2)	0.7
H31A	0.2861	0.8217	0.4106	0.109*	0.7
H31B	0.2541	0.8279	0.5100	0.109*	0.7
C32	0.4329 (7)	0.9221 (5)	0.4177 (4)	0.0885 (16)	0.7
H32A	0.4131	0.9731	0.4533	0.106*	0.7
H32B	0.4428	0.9688	0.3528	0.106*	0.7
C33	0.5647 (8)	0.8600 (4)	0.4263 (4)	0.0668 (18)	0.7
H33	0.6439	0.9103	0.4219	0.080*	0.7
C34	0.5009 (6)	0.7574 (5)	0.5187 (4)	0.0837 (15)	0.7
C35	0.6079 (9)	0.6655 (6)	0.5374 (6)	0.0905 (19)	0.7
H35A	0.6458	0.6447	0.4837	0.136*	0.7
H35B	0.6821	0.7005	0.5486	0.136*	0.7
H35C	0.5614	0.5951	0.5913	0.136*	0.7
C36	0.4387 (7)	0.7926 (5)	0.6011 (4)	0.0886 (17)	0.7
H36A	0.4006	0.7223	0.6570	0.133*	0.7
H36B	0.5099	0.8370	0.6083	0.133*	0.7
H36C	0.3660	0.8412	0.5908	0.133*	0.7
C37	0.2956 (15)	0.5992 (10)	0.5560 (9)	0.090 (4)	0.7
H37A	0.2383	0.6140	0.6094	0.135*	0.7
H37B	0.2375	0.5779	0.5259	0.135*	0.7
H37C	0.3485	0.5351	0.5761	0.135*	0.7
C30'	0.4475 (12)	0.6790 (9)	0.5002 (8)	0.066 (3)*	0.3
C31'	0.5572 (17)	0.6658 (16)	0.5409 (15)	0.094 (7)*	0.3
H31C	0.6026	0.5967	0.5366	0.112*	0.3
H31D	0.5182	0.6539	0.6066	0.112*	0.3
C32'	0.6593 (13)	0.7734 (11)	0.4905 (9)	0.083 (3)*	0.3
H32C	0.7465	0.7579	0.4529	0.100*	0.3
H32D	0.6775	0.8055	0.5333	0.100*	0.3
C33'	0.5827 (19)	0.857 (2)	0.4286 (17)	0.146 (14)*	0.3
H33'	0.6111	0.9433	0.4052	0.176*	0.3
C34'	0.4308 (12)	0.8135 (10)	0.4799 (8)	0.076 (3)*	0.3
C35'	0.3305 (15)	0.8611 (14)	0.4200 (11)	0.082 (4)*	0.3
H35D	0.2358	0.8285	0.4584	0.122*	0.3
H35E	0.3387	0.9466	0.3979	0.122*	0.3
H35F	0.3562	0.8372	0.3674	0.122*	0.3
C36'	0.372 (3)	0.842 (2)	0.5673 (14)	0.162 (9)*	0.3
H36D	0.2743	0.8113	0.5965	0.243*	0.3
H36E	0.4207	0.8056	0.6105	0.243*	0.3
H36F	0.3821	0.9267	0.5502	0.243*	0.3
C37'	0.313 (3)	0.598 (3)	0.553 (3)	0.105 (12)*	0.3
H37D	0.2512	0.6176	0.5170	0.157*	0.3
H37E	0.3313	0.5166	0.5649	0.157*	0.3
H37F	0.2708	0.6064	0.6119	0.157*	0.3

### Atomic displacement parameters (Å^2^)

**Table:**

	*U*^11^	*U*^22^	*U*^33^	*U*^12^	*U*^13^	*U*^23^
Cd1	0.04595 (11)	0.03978 (11)	0.07460 (14)	0.00295 (7)	−0.02207 (9)	−0.02640 (9)
S1	0.0592 (4)	0.0487 (4)	0.0981 (5)	0.0148 (3)	−0.0396 (4)	−0.0376 (4)
O1	0.0486 (10)	0.0435 (10)	0.0958 (15)	0.0036 (7)	−0.0252 (10)	−0.0300 (10)
N1	0.0618 (13)	0.0424 (11)	0.0850 (16)	0.0137 (10)	−0.0352 (12)	−0.0322 (11)
N2	0.0561 (12)	0.0380 (10)	0.0741 (14)	0.0058 (9)	−0.0255 (11)	−0.0249 (10)
N3	0.0477 (11)	0.0392 (10)	0.0663 (13)	0.0036 (8)	−0.0225 (10)	−0.0215 (9)
C1	0.0587 (14)	0.0373 (12)	0.0648 (15)	0.0055 (10)	−0.0254 (12)	−0.0195 (11)
C2	0.0571 (14)	0.0411 (13)	0.0662 (16)	0.0043 (10)	−0.0202 (12)	−0.0244 (12)
C3	0.0570 (15)	0.0406 (13)	0.0762 (18)	0.0060 (11)	−0.0214 (13)	−0.0238 (12)
C4	0.0634 (17)	0.0457 (15)	0.096 (2)	0.0032 (12)	−0.0216 (16)	−0.0333 (15)
C5	0.0663 (18)	0.0607 (18)	0.097 (2)	0.0000 (14)	−0.0265 (17)	−0.0431 (17)
C6	0.0709 (19)	0.0677 (19)	0.091 (2)	0.0112 (15)	−0.0385 (17)	−0.0405 (17)
C7	0.0753 (19)	0.0495 (15)	0.083 (2)	0.0152 (13)	−0.0383 (16)	−0.0303 (14)
C8	0.0455 (13)	0.0448 (13)	0.0712 (16)	0.0021 (10)	−0.0221 (12)	−0.0233 (12)
C9	0.0456 (13)	0.0438 (13)	0.0770 (17)	0.0032 (10)	−0.0207 (12)	−0.0232 (12)
C10	0.0458 (14)	0.0570 (16)	0.094 (2)	0.0066 (12)	−0.0229 (14)	−0.0315 (16)
C11	0.0594 (17)	0.079 (2)	0.081 (2)	0.0105 (15)	−0.0315 (16)	−0.0179 (17)
C12	0.0640 (19)	0.100 (3)	0.086 (2)	0.0117 (18)	−0.0366 (17)	−0.040 (2)
C13	0.0504 (14)	0.0573 (16)	0.092 (2)	0.0010 (12)	−0.0253 (14)	−0.0355 (15)
C14	0.0498 (15)	0.0633 (18)	0.089 (2)	−0.0008 (13)	−0.0205 (15)	−0.0284 (16)
C15	0.067 (2)	0.098 (3)	0.071 (2)	−0.0088 (18)	−0.0145 (16)	−0.0210 (19)
C16	0.0473 (16)	0.091 (3)	0.123 (3)	−0.0046 (16)	−0.0214 (18)	−0.043 (2)
C17	0.0560 (16)	0.068 (2)	0.111 (3)	0.0188 (14)	−0.0273 (17)	−0.0414 (19)
S21	0.0521 (3)	0.0459 (3)	0.0736 (4)	−0.0015 (3)	−0.0189 (3)	−0.0286 (3)
O21	0.0807 (14)	0.0451 (10)	0.0729 (13)	−0.0001 (9)	−0.0272 (11)	−0.0230 (9)
N21	0.0493 (11)	0.0479 (12)	0.0795 (15)	−0.0034 (9)	−0.0151 (11)	−0.0337 (11)
N22	0.0496 (11)	0.0464 (12)	0.0762 (15)	0.0013 (9)	−0.0206 (11)	−0.0285 (11)
N23	0.0523 (12)	0.0435 (11)	0.0723 (15)	0.0055 (9)	−0.0233 (11)	−0.0261 (10)
C21	0.0419 (12)	0.0455 (13)	0.0802 (18)	0.0071 (10)	−0.0238 (12)	−0.0295 (13)
C22	0.0412 (12)	0.0559 (15)	0.102 (2)	0.0079 (11)	−0.0258 (14)	−0.0462 (16)
C23	0.0588 (16)	0.0474 (15)	0.116 (3)	0.0086 (12)	−0.0411 (17)	−0.0369 (16)
C24	0.071 (2)	0.0524 (17)	0.162 (4)	0.0172 (15)	−0.061 (2)	−0.055 (2)
C25	0.0628 (19)	0.084 (3)	0.187 (5)	0.0211 (18)	−0.054 (3)	−0.096 (3)
C26	0.064 (2)	0.129 (4)	0.155 (4)	0.000 (2)	−0.024 (2)	−0.109 (4)
C27	0.0594 (18)	0.097 (3)	0.113 (3)	−0.0099 (17)	−0.0136 (18)	−0.071 (2)
C28	0.0721 (18)	0.0455 (14)	0.0690 (18)	0.0020 (12)	−0.0210 (15)	−0.0255 (13)
C29	0.085 (2)	0.0455 (15)	0.0646 (17)	−0.0009 (13)	−0.0239 (15)	−0.0195 (13)
C30	0.070 (3)	0.056 (2)	0.065 (3)	0.001 (2)	−0.023 (2)	−0.023 (2)
C31	0.118 (5)	0.080 (4)	0.061 (3)	0.037 (4)	−0.020 (3)	−0.020 (3)
C32	0.121 (5)	0.066 (3)	0.089 (4)	0.025 (3)	−0.041 (3)	−0.036 (3)
C33	0.096 (4)	0.042 (2)	0.060 (3)	−0.014 (2)	−0.014 (2)	−0.0268 (19)
C34	0.109 (4)	0.075 (3)	0.074 (3)	−0.001 (3)	−0.031 (3)	−0.036 (3)
C35	0.098 (5)	0.083 (4)	0.101 (5)	0.021 (4)	−0.052 (4)	−0.032 (3)
C36	0.121 (5)	0.077 (3)	0.068 (3)	−0.006 (3)	−0.020 (3)	−0.037 (3)
C37	0.119 (8)	0.061 (4)	0.066 (4)	−0.030 (4)	−0.002 (4)	−0.023 (3)

### Geometric parameters (Å, º)

**Table:**

Cd1—N3	2.306 (2)	C22—C23	1.394 (5)
Cd1—N23	2.318 (2)	C23—C24	1.390 (4)
Cd1—S1	2.5245 (7)	C23—H23	0.9500
Cd1—S21	2.5445 (7)	C24—C25	1.362 (7)
Cd1—O1	2.5839 (19)	C24—H24	0.9500
Cd1—O21	2.627 (2)	C25—C26	1.377 (7)
S1—C1	1.734 (3)	C25—H25	0.9500
O1—C9	1.219 (3)	C26—C27	1.403 (5)
N1—C1	1.364 (3)	C26—H26	0.9500
N1—C2	1.414 (3)	C27—H27	0.9500
N1—H1	0.8800	C28—C29	1.484 (4)
N2—C1	1.319 (3)	C28—C33	1.492 (6)
N2—N3	1.362 (3)	C28—C33'	1.52 (3)
N3—C8	1.280 (3)	C29—C30'	1.491 (12)
C2—C7	1.390 (4)	C29—C30	1.550 (6)
C2—C3	1.398 (4)	C30—C37	1.500 (7)
C3—C4	1.381 (4)	C30—C31	1.553 (7)
C3—H3	0.9500	C30—C34	1.569 (7)
C4—C5	1.375 (5)	C31—C32	1.463 (9)
C4—H4	0.9500	C31—H31A	0.9900
C5—C6	1.377 (5)	C31—H31B	0.9900
C5—H5	0.9500	C32—C33	1.585 (11)
C6—C7	1.387 (4)	C32—H32A	0.9900
C6—H6	0.9500	C32—H32B	0.9900
C7—H7	0.9500	C33—C34	1.536 (7)
C8—C9	1.485 (4)	C33—H33	1.0000
C8—C13	1.503 (4)	C34—C36	1.535 (7)
C9—C10	1.511 (4)	C34—C35	1.603 (9)
C10—C17	1.506 (4)	C35—H35A	0.9800
C10—C14	1.542 (4)	C35—H35B	0.9800
C10—C11	1.591 (5)	C35—H35C	0.9800
C11—C12	1.521 (5)	C36—H36A	0.9800
C11—H11A	0.9900	C36—H36B	0.9800
C11—H11B	0.9900	C36—H36C	0.9800
C12—C13	1.574 (5)	C37—H37A	0.9800
C12—H12A	0.9900	C37—H37B	0.9800
C12—H12B	0.9900	C37—H37C	0.9800
C13—C14	1.536 (5)	C30'—C31'	1.469 (15)
C13—H13	1.0000	C30'—C37'	1.529 (16)
C14—C16	1.531 (4)	C30'—C34'	1.595 (13)
C14—C15	1.537 (5)	C31'—C32'	1.499 (17)
C15—H15A	0.9800	C31'—H31C	0.9900
C15—H15B	0.9800	C31'—H31D	0.9900
C15—H15C	0.9800	C32'—C33'	1.58 (2)
C16—H16A	0.9800	C32'—H32C	0.9900
C16—H16B	0.9800	C32'—H32D	0.9900
C16—H16C	0.9800	C33'—C34'	1.530 (16)
C17—H17A	0.9800	C33'—H33'	1.0000
C17—H17B	0.9800	C34'—C36'	1.553 (15)
C17—H17C	0.9800	C34'—C35'	1.619 (14)
S21—C21	1.743 (3)	C35'—H35D	0.9800
O21—C29	1.219 (4)	C35'—H35E	0.9800
N21—C21	1.365 (3)	C35'—H35F	0.9800
N21—C22	1.415 (3)	C36'—H36D	0.9800
N21—H21	0.8800	C36'—H36E	0.9800
N22—C21	1.313 (4)	C36'—H36F	0.9800
N22—N23	1.367 (3)	C37'—H37D	0.9800
N23—C28	1.278 (4)	C37'—H37E	0.9800
C22—C27	1.373 (5)	C37'—H37F	0.9800
			
N3—Cd1—N23	141.00 (8)	C25—C24—H24	120.2
N3—Cd1—S1	75.51 (5)	C23—C24—H24	120.2
N23—Cd1—S1	129.89 (6)	C24—C25—C26	119.8 (3)
N3—Cd1—S21	131.35 (6)	C24—C25—H25	120.1
N23—Cd1—S21	74.79 (6)	C26—C25—H25	120.1
S1—Cd1—S21	107.49 (3)	C25—C26—C27	121.4 (4)
N3—Cd1—O1	69.93 (7)	C25—C26—H26	119.3
N23—Cd1—O1	79.45 (7)	C27—C26—H26	119.3
S1—Cd1—O1	145.35 (4)	C22—C27—C26	118.6 (4)
S21—Cd1—O1	97.17 (5)	C22—C27—H27	120.7
N3—Cd1—O21	79.09 (7)	C26—C27—H27	120.7
N23—Cd1—O21	69.40 (7)	N23—C28—C29	119.2 (3)
S1—Cd1—O21	97.73 (5)	N23—C28—C33	134.7 (3)
S21—Cd1—O21	144.07 (5)	C29—C28—C33	105.5 (3)
O1—Cd1—O21	73.80 (7)	N23—C28—C33'	132.2 (7)
C1—S1—Cd1	97.71 (9)	C29—C28—C33'	108.6 (7)
C9—O1—Cd1	107.48 (17)	O21—C29—C28	125.9 (3)
C1—N1—C2	130.3 (2)	O21—C29—C30'	128.8 (5)
C1—N1—H1	114.8	C28—C29—C30'	102.4 (5)
C2—N1—H1	114.8	O21—C29—C30	127.9 (3)
C1—N2—N3	113.5 (2)	C28—C29—C30	105.5 (3)
C8—N3—N2	118.0 (2)	C37—C30—C29	115.9 (6)
C8—N3—Cd1	117.85 (17)	C37—C30—C31	117.2 (8)
N2—N3—Cd1	123.77 (16)	C29—C30—C31	105.7 (4)
N2—C1—N1	117.3 (2)	C37—C30—C34	120.1 (7)
N2—C1—S1	129.2 (2)	C29—C30—C34	97.8 (4)
N1—C1—S1	113.5 (2)	C31—C30—C34	96.8 (4)
C7—C2—C3	119.2 (3)	C32—C31—C30	109.5 (5)
C7—C2—N1	124.1 (2)	C32—C31—H31A	109.8
C3—C2—N1	116.6 (3)	C30—C31—H31A	109.8
C4—C3—C2	120.1 (3)	C32—C31—H31B	109.8
C4—C3—H3	119.9	C30—C31—H31B	109.8
C2—C3—H3	119.9	H31A—C31—H31B	108.2
C5—C4—C3	120.5 (3)	C31—C32—C33	101.7 (4)
C5—C4—H4	119.7	C31—C32—H32A	111.4
C3—C4—H4	119.7	C33—C32—H32A	111.4
C4—C5—C6	119.6 (3)	C31—C32—H32B	111.4
C4—C5—H5	120.2	C33—C32—H32B	111.4
C6—C5—H5	120.2	H32A—C32—H32B	109.3
C5—C6—C7	121.0 (3)	C28—C33—C34	103.4 (3)
C5—C6—H6	119.5	C28—C33—C32	104.1 (5)
C7—C6—H6	119.5	C34—C33—C32	99.7 (5)
C6—C7—C2	119.5 (3)	C28—C33—H33	115.8
C6—C7—H7	120.2	C34—C33—H33	115.8
C2—C7—H7	120.2	C32—C33—H33	115.8
N3—C8—C9	118.9 (2)	C36—C34—C33	114.8 (4)
N3—C8—C13	135.2 (2)	C36—C34—C30	113.5 (5)
C9—C8—C13	105.8 (2)	C33—C34—C30	95.9 (4)
O1—C9—C8	125.3 (2)	C36—C34—C35	111.4 (5)
O1—C9—C10	129.5 (3)	C33—C34—C35	110.7 (6)
C8—C9—C10	105.2 (2)	C30—C34—C35	109.6 (5)
C17—C10—C9	115.7 (3)	C34—C35—H35A	109.5
C17—C10—C14	120.2 (3)	C34—C35—H35B	109.5
C9—C10—C14	100.2 (2)	H35A—C35—H35B	109.5
C17—C10—C11	114.9 (3)	C34—C35—H35C	109.5
C9—C10—C11	103.0 (3)	H35A—C35—H35C	109.5
C14—C10—C11	100.1 (3)	H35B—C35—H35C	109.5
C12—C11—C10	105.2 (3)	C34—C36—H36A	109.5
C12—C11—H11A	110.7	C34—C36—H36B	109.5
C10—C11—H11A	110.7	H36A—C36—H36B	109.5
C12—C11—H11B	110.7	C34—C36—H36C	109.5
C10—C11—H11B	110.7	H36A—C36—H36C	109.5
H11A—C11—H11B	108.8	H36B—C36—H36C	109.5
C11—C12—C13	103.0 (3)	C30—C37—H37A	109.5
C11—C12—H12A	111.2	C30—C37—H37B	109.5
C13—C12—H12A	111.2	H37A—C37—H37B	109.5
C11—C12—H12B	111.2	C30—C37—H37C	109.5
C13—C12—H12B	111.2	H37A—C37—H37C	109.5
H12A—C12—H12B	109.1	H37B—C37—H37C	109.5
C8—C13—C14	100.9 (2)	C31'—C30'—C29	116.5 (12)
C8—C13—C12	104.5 (3)	C31'—C30'—C37'	120 (2)
C14—C13—C12	101.1 (3)	C29—C30'—C37'	110.4 (19)
C8—C13—H13	116.0	C31'—C30'—C34'	100.2 (11)
C14—C13—H13	116.0	C29—C30'—C34'	92.6 (7)
C12—C13—H13	116.0	C37'—C30'—C34'	113.9 (18)
C16—C14—C13	114.2 (3)	C30'—C31'—C32'	109.4 (13)
C16—C14—C15	109.6 (3)	C30'—C31'—H31C	109.8
C13—C14—C15	111.9 (3)	C32'—C31'—H31C	109.8
C16—C14—C10	112.9 (3)	C30'—C31'—H31D	109.8
C13—C14—C10	96.3 (2)	C32'—C31'—H31D	109.8
C15—C14—C10	111.6 (3)	H31C—C31'—H31D	108.2
C14—C15—H15A	109.5	C31'—C32'—C33'	101.2 (11)
C14—C15—H15B	109.5	C31'—C32'—H32C	111.5
H15A—C15—H15B	109.5	C33'—C32'—H32C	111.5
C14—C15—H15C	109.5	C31'—C32'—H32D	111.5
H15A—C15—H15C	109.5	C33'—C32'—H32D	111.5
H15B—C15—H15C	109.5	H32C—C32'—H32D	109.4
C14—C16—H16A	109.5	C28—C33'—C34'	94.1 (14)
C14—C16—H16B	109.5	C28—C33'—C32'	101.9 (17)
H16A—C16—H16B	109.5	C34'—C33'—C32'	105.0 (13)
C14—C16—H16C	109.5	C28—C33'—H33'	117.5
H16A—C16—H16C	109.5	C34'—C33'—H33'	117.5
H16B—C16—H16C	109.5	C32'—C33'—H33'	117.5
C10—C17—H17A	109.5	C33'—C34'—C36'	114.0 (15)
C10—C17—H17B	109.5	C33'—C34'—C30'	95.3 (12)
H17A—C17—H17B	109.5	C36'—C34'—C30'	114.4 (12)
C10—C17—H17C	109.5	C33'—C34'—C35'	115.3 (12)
H17A—C17—H17C	109.5	C36'—C34'—C35'	105.7 (12)
H17B—C17—H17C	109.5	C30'—C34'—C35'	112.3 (10)
C21—S21—Cd1	98.24 (10)	C34'—C35'—H35D	109.5
C29—O21—Cd1	106.48 (19)	C34'—C35'—H35E	109.5
C21—N21—C22	131.4 (3)	H35D—C35'—H35E	109.5
C21—N21—H21	114.3	C34'—C35'—H35F	109.5
C22—N21—H21	114.3	H35D—C35'—H35F	109.5
C21—N22—N23	113.8 (2)	H35E—C35'—H35F	109.5
C28—N23—N22	116.9 (2)	C34'—C36'—H36D	109.5
C28—N23—Cd1	118.48 (18)	C34'—C36'—H36E	109.5
N22—N23—Cd1	124.37 (18)	H36D—C36'—H36E	109.5
N22—C21—N21	117.9 (2)	C34'—C36'—H36F	109.5
N22—C21—S21	128.8 (2)	H36D—C36'—H36F	109.5
N21—C21—S21	113.3 (2)	H36E—C36'—H36F	109.5
C27—C22—C23	119.6 (3)	C30'—C37'—H37D	109.5
C27—C22—N21	125.1 (3)	C30'—C37'—H37E	109.5
C23—C22—N21	115.3 (3)	H37D—C37'—H37E	109.5
C24—C23—C22	120.9 (4)	C30'—C37'—H37F	109.5
C24—C23—H23	119.5	H37D—C37'—H37F	109.5
C22—C23—H23	119.5	H37E—C37'—H37F	109.5
C25—C24—C23	119.6 (4)		

### Hydrogen-bond geometry (Å, º)

**Table:**

*D*—H···*A*	*D*—H	H···*A*	*D*···*A*	*D*—H···*A*
N21—H21···S21^i^	0.88	2.58	3.363 (3)	148
C23—H23···S21^i^	0.95	2.97	3.629 (4)	128

Symmetry code: (i) −*x*+2, −*y*+2, −*z*.
