# Supplementary figures and images for: Crystal structure of cis-bis­{4-phenyl-1-[(3R)-1,7,7-tri­methyl-2-oxobi­cyclo­[2.2.1]heptan-3-ylidene]thio­semicarbazidato-κ3 O,N 1,S}cadmium(II) with an unknown solvent mol­ecule
Source: Acta Crystallogr E Crystallogr Commun. 2015 Nov 21;71(Pt 12):m234–5. doi: 10.1107/S2056989015021428 (PMC4719850; doi:10.1107/S2056989015021428)

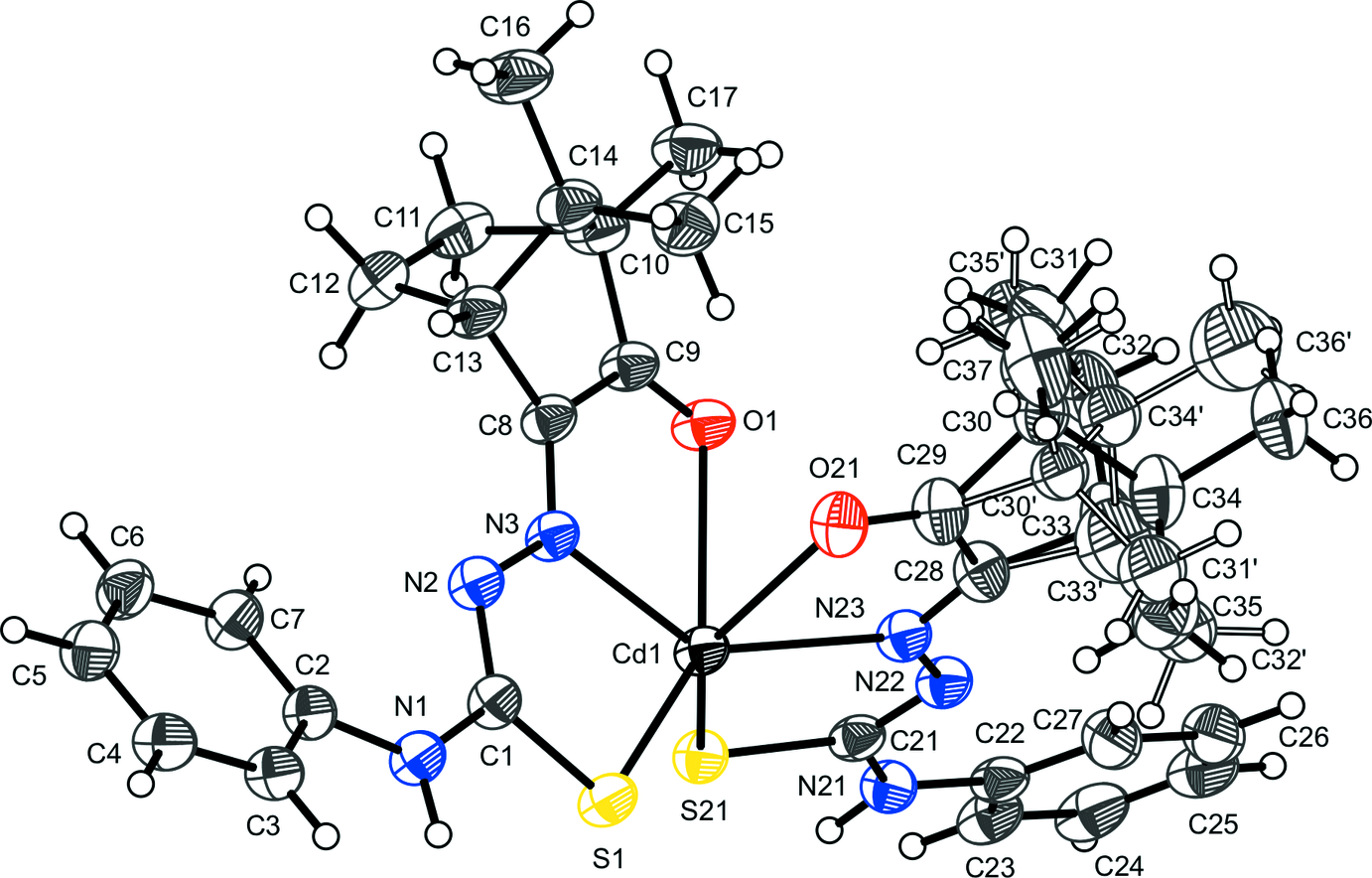

Supplement: Supplementary file 3 [file e-71-0m234-fig1.tif]

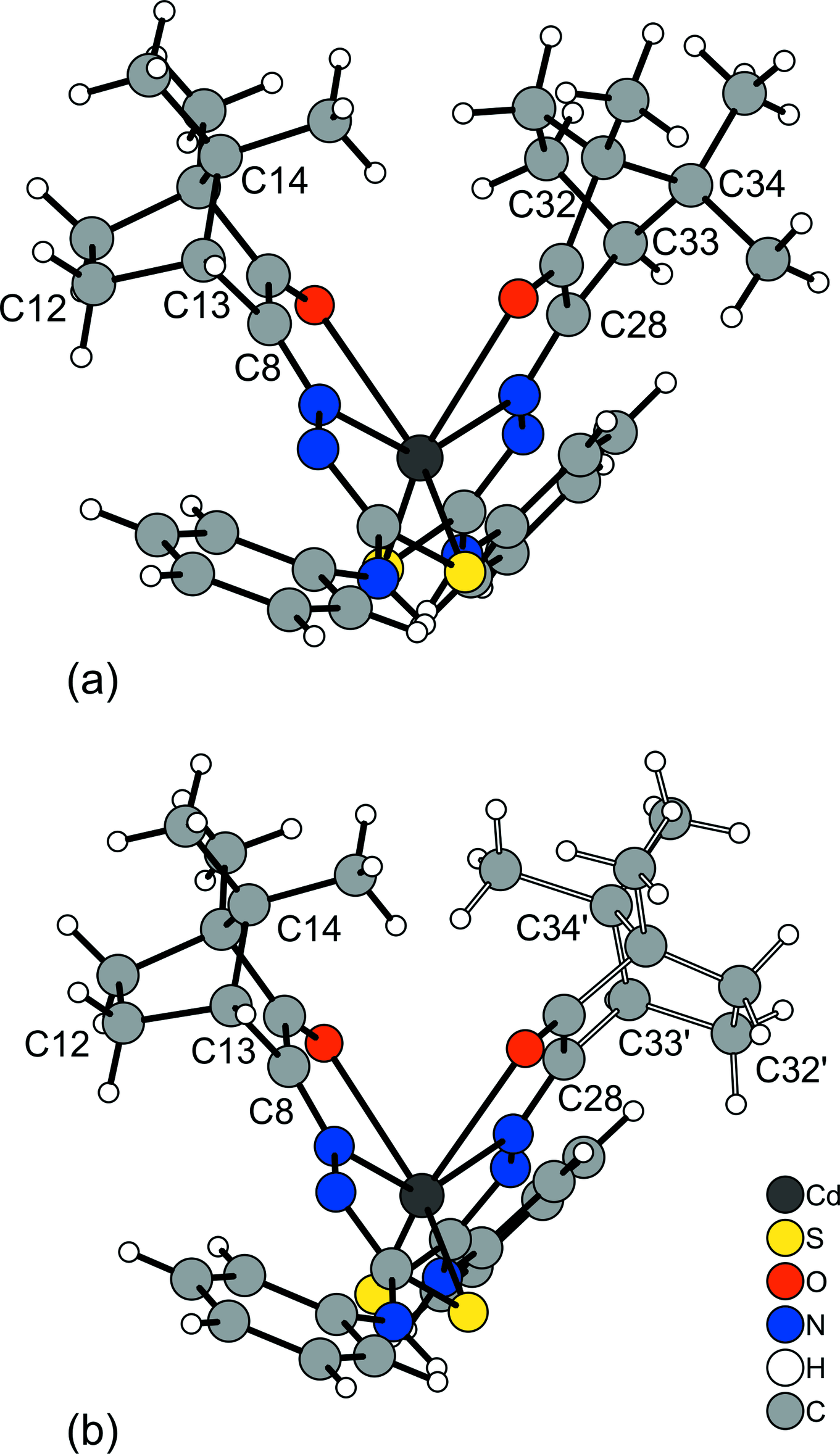

Supplement: Supplementary file 4 [file e-71-0m234-fig2.tif]

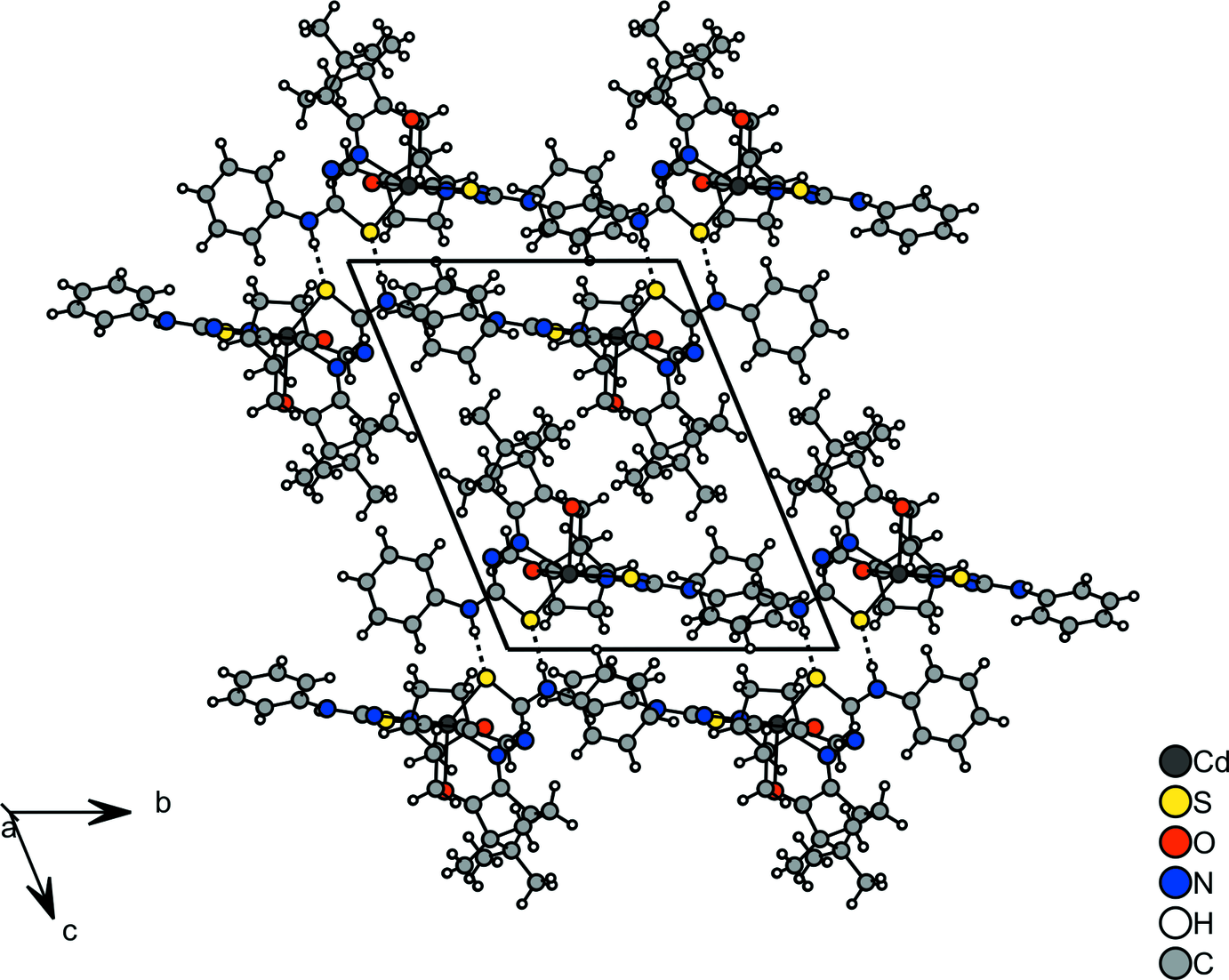

Supplement: Supplementary file 5 [file e-71-0m234-fig3.tif]
